# Natural Isoforms of *Listeria monocytogenes* Virulence Factor Inlb Differ in c-Met Binding Efficiency and Differently Affect Uptake and Survival Listeria in Macrophage †

**DOI:** 10.3390/ijms24087256

**Published:** 2023-04-14

**Authors:** Yaroslava M. Chalenko, Daria A. Slonova, Olga I. Kechko, Egor V. Kalinin, Vladimir A. Mitkevich, Svetlana A. Ermolaeva

**Affiliations:** 1 Laboratory of Ecology of Pathogenic Bacteria, Gamaleya Research Center of Epidemiology and Microbiology, 123098 Moscow, Russia; kalinin.egor@bk.ru; 2 Laboratory of Metagenome Analysis, Skolkovo Institute of Science and Technology, 121205 Moscow, Russia; darya.romanenko@phystech.edu; 3 Laboratory of Conformational Polymorphism of Proteins in Health and Disease, Engelhardt Institute of Molecular Biology, Russian Academy of Sciences, 119991 Moscow, Russia; olga.kechko@gmail.com (O.I.K.); mitkevich@gmail.com (V.A.M.)

**Keywords:** listeriosis, *Listeria monocytogenes*, InlB, c-Met, gC1qR, M1 macrophages

## Abstract

*Listeria monocytogenes* virulence factor InlB specifically interacts with the receptors c-Met and gC1q-R. Both receptors are present in non-professional and professional phagocytes, including macrophages. Phylogenetically defined InlB isoforms differently support invasion into non-professional phagocytes. This work deals with the effects of InlB isoforms on *L. monocytogenes* uptake and intracellular proliferation in human macrophages. Three isoforms of the receptor binding domain (idInlB) were derived from phylogenetically distinct *L. monocytogenes* strains belonging to the highly virulent CC1 (idInlB_CC1_), medium-virulence CC7 (idInlB_CC7_), and low-virulence CC9 (idInlB_CC9_) clonal complexes. The constant dissociation increased in the order idInlB_CC1_ << idInlB_CC7_ < idInlB_CC9_ for interactions with c-Met, and idInlB_CC1_ ≈ idInlB_CC7_ < idInlB_CC9_ for interactions with gC1q-R. The comparison of uptake and intracellular proliferation of isogenic recombinant strains which expressed full-length InlBs revealed that the strain expressing idInlB_CC1_ proliferated in macrophages twice as efficiently as other strains. Macrophage pretreatment with idInlB_CC1_ followed by recombinant *L. monocytogenes* infection disturbed macrophage functions decreasing pathogen uptake and improving its intracellular multiplication. Similar pretreatment with idInlB_CC7_ decreased bacterial uptake but also impaired intracellular multiplication. The obtained results demonstrated that InlB impaired macrophage functions in an idInlB isoform-dependent manner. These data suggest a novel InlB function in *L. monocytogenes* virulence.

## 1. Introduction

The Gram-positive bacterium *Listeria monocytogenes* causes a severe foodborne disease, listeriosis [1]. The species *L. monocytogenes* is divided into four phylogenetic lineages and multiple clonal complexes, which are highly diverse in their epidemic potential [2,3,4]. Lineage I strains are responsible for the majority of outbreaks and at least half of sporadic cases of listeriosis in humans and animals [5,6]. Clonal complexes CC1, CC2, CC4, and CC6, belonging to lineage I, are hypervirulent in humans [7]. Lineage II strains are responsible for the majority of other listeriosis cases in humans and are overrepresented among animal and food isolates [5,7]. The clonal complex CC7, belonging to lineage II, is considered of medium virulence but it is widespread in natural foci of infection in Eurasia and is often found in food products, which results in its high frequency in human listeriosis in Russia and some other countries [8,9]. Strains of lineages III and IV are rare among human and animal isolates [10].

The discrepancy between the availability of major virulence factors and differences in the epidemic potential suggest specific mechanisms providing the formation of hypervirulent clones. The possession of additional virulence factors is one such mechanism [7]. Another mechanism is the natural variability of the major virulence factors typical for phylogenetically distinct *L. monocytogenes* clones [11,12,13]. Such variability seems to be important in the evolution of hypervirulent strains, as well as playing an important role in the formation of low-virulence strains [14,15]. Certain allelic variants of the major virulence factors, such as the phospholipase PlcA or invasins of the internalin family InlA and InlB, supply higher virulence, at least to strains that carry these variants [13,16,17]. The introduction of allelic variants of individual virulence genes from the highly virulent strain to low-virulence strains restored their virulence in mice [16]. Natural isoforms of the major invasion factor InlB, when they are placed into the same genetic background, affect *L. monocytogenes* interactions differently with epithelial cells derived from humans and mammals, including mice, sheep, and bats, and its virulence in mice [13,18,19,20].

InlB specifically interacts with mammalian cell surface target receptors c-Met (mesenchymal-epithelial transition factor gene) and gC1qR to mediate *L. monocytogenes* active invasion into epithelial cells and some other types of non-professional phagocytes [21,22,23]. The amino-terminal InlB domain (internalin domain, idInlB), which includes the leucine-rich repeat (LRR) domain flanked by cap- and immunoglobulin-like domains, directly interacts with both receptors [23,24]. The carboxy-terminal GW domains non-covalently bind InlB to the bacterial surface, so InlB is present in two forms, secreted and surface-bound, both of which interact with the target receptors.

c-Met belongs to the family of tyrosine-kinase receptors controlling cell proliferation and migration in response to the binding of soluble growth factors [25]. InlB causes c-Met dimerization and autophosphorylation in a similar way as happens upon c-Met interactions with its physiological ligand HGF (hepatocyte growth factor) [26,27]. Application of purified idInlB to epithelial cells induces the activation of Erk1/2 and Akt kinase-specific intracellular signaling pathways [26]. The kinetics of Erk1/2 and Akt kinase phosphorylation are diverse, depending on the idInlB isoform and differing noticeably for idInlBs derived from strains belonging to different phylogenetic lineages [13]. Another InlB target receptor, gC1qR, was first identified as a receptor of the C1q complement system, but later, it was shown to bind various proteins found in plasma, on cell surfaces, and on pathogenic microorganisms [21,28]. gC1qR differentially interacts with phylogenetically diverse idInlBs [13]. In particular, the antibody against gC1q-R amino acids 221–249 inhibited invasion of the *L. monocytogenes* strain carrying lineage I idInlB but not isogenic strains carrying lineage II idInlB [13].

Both receptors, c-Met and gC1q-R, are found in different cell types including not only epithelial cells and other non-professional phagocytes but also cells of the immune system. In particular, both receptors were found on the surface of macrophages, which are professional phagocytes that provide a first line of innate immune defense against invasive pathogens [21,25,28,29,30,31]. Macrophages are crucial for clearing *L. monocytogenes* infection and establishing immunity to secondary infection with this pathogen [32,33]. The fate of *L. monocytogenes* in macrophages is not predetermined but depends on the macrophage status. In non-activated macrophages, *L. monocytogenes* escapes from the phagosome and multiplies within the cytoplasm while activated macrophages generate reactive oxygen or nitrogen intermediates early after bacterial uptake that effectively kill the bacteria [34]. *L. monocytogenes* exploits host-cell structures and mechanisms such as the host cytosolic cysteine protease calpain or the type I IFN response receptor to facilitate its escape from the phagosome and survival inside macrophages [35,36]. Bacterial virulence factors are important for the interplay between the bacterium and macrophages. The thiol-dependent hemolysin LLO, which provides phagosome escaping, is critically important for survival in macrophages [37]. The O-acetyltransferase OatA, which modifies *L. monocytogenes* peptidoglycan to confer resistance to different types of antimicrobial compounds targeting the bacterial cell wall, is required for Listeria growth in macrophages [38]. However, little is known about the control of macrophage intracellular signaling and response by bacterial factors. The role of InlB has been thoroughly studied in interactions of *L. monocytogenes* with epithelial cells; at the same time, it is poorly described when interactions with macrophages are considered.

Recently, the authors of the present paper demonstrated that InlB affected *L. monocytogenes* uptake and multiplication within M1 macrophages [39]. This work focused on whether InlB interactions with the extracellular receptors affect *L. monocytogenes* intracellular survival and multiplication in macrophages. Next, the role of phylogenetically determined variability in InlB influence on *L. monocytogenes* interactions with macrophages was determined. Phylogenetically determined effects were found in interactions between *L. monocytogenes* and professional phagocytes, which could potentially explain the high virulent potential of individual clonal complexes.

## 2. Results

### 2.1. idInlB Isoforms Characteristic for Lineage I (Clonal Complex CC1) and Lineage II (Clonal complexes CC7 and CC9) Differently Bind c-Met but not gC1qR

The efficiency of interactions of distinct InlB isoforms typical for *L. monocytogenes* lineage I and lineage II strains with the InlB target receptors c-Met and gC1qR was compared. Three purified proteins representing the receptor-binding InlB domain idInlB (idInlB_CC1_, idInlB_CC7_and idInlB_CC9_) were used. The comprehensive study of *L. monocytogenes* strains of food and clinical origin performed by Maury and colleagues (2016) revealed strong association of certain clonal groups (CC1, CC2, CC4 and CC6) with a clinical origin (*p* < 1 × 10^−4^; 62% of strains from these clones were of clinical origin). These clones might be considered highly virulent, while other clones, which are strongly associated with a food origin (CC121 and CC9; *p* < 1 × 10^−4^; only 10.3% were of clinical origin) can be considered low virulence. Clones with intermediate characteristics (CC7) can be considered as of medium virulence. The isoform idInlB_CC1_ was found merely in strains of the hypervirulent clonal complex CC1 belonging to lineage I. The isoform idInlB_CC7_ is typical for the CC7 and a few other lineage II clonal complexes including CC19, CC21, CC177, and some others [8,9,15,40,41]. The isoform idInlB_CC9_ is typical for the low-virulence CC9, which includes the type *L. monocytogenes* strain EGDe [7]. idInlB_CC1_, idInlB_CC7_, and idInlB_CC9_ are 321 aa long and differ by 13 amino acids, with idInlB_CC1_ differing from both idInlB_CC7_and idInlB_CC9_, and four amino acid substitutions, with idInlB_CC7_ and idInlB_CC9_ differing within the lineage II (Supplementary Material Figure S1).

**Figure 1:**
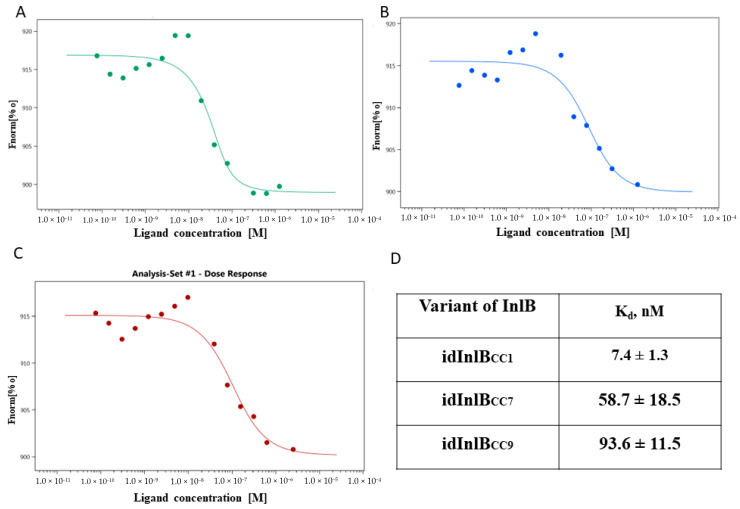
MST binding curves of idInlB_CC1_ (**A**), idInlB_CC7_ (**B**) and idInlB_CC9_ (**C**) with c-Met. The c-Met were titrated against fluorescent labeled 50 nM idInlB variants. The normalized thermophoresis value Fnorm% was plotted against the c-Met concentration. Data of one from three independent measurements are shown. (**D**) Mean values of dissociation constants (K_d_) ± standard deviation for human c-Met binding with InlB variants from three independent experiments.

To characterize the interactions of idInlBs with human receptors, dissociation constants were determined using MST technology [42,43]. The c-Met extracellular domain, which was a part of the soluble chimera protein, was titrated in a concentration range from 0.076 nM to 2.5 μM against fluorescent-labeled 50 nM isoforms of idInlB. The normalized thermophoresis value Fnorm% plotted against the idInlB_CC1_, idInlB_CC7_ and idInlB_CC9_ concentration fitted to a 1:1 binding model resulting in a dissociation constant Kd = 7.4 ± 1.3 nM, 58.7 ± 18.5 nM and 93.6 ± 11.5 nM, respectively (Figure 1A–D). These data showed that three variants bound the receptor, but the idInlB_CC1_ was bound eight times stronger than idInlB_CC7_ and 12 times stronger than idInlB_CC9_.

**Figure 2:**
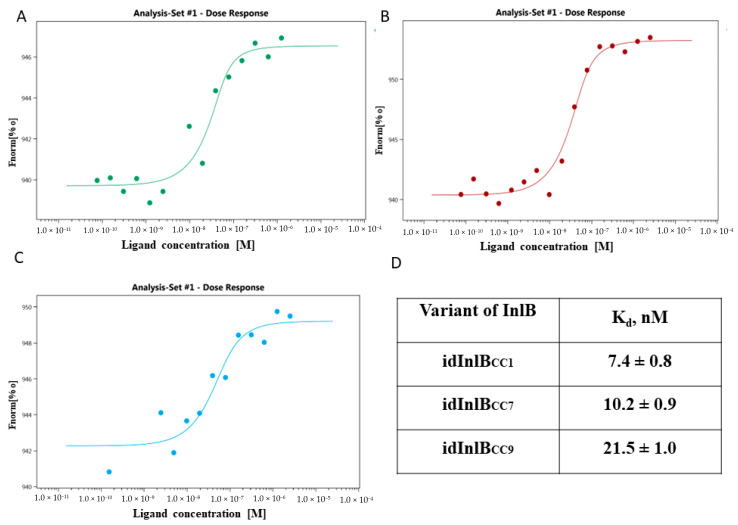
MST binding curves of idInlB_CC1_ (**A**), idInlB_CC7_ (**B**) and idInlB_CC9_ (**C**) with gC1qR. The gC1qR were titrated against fluorescent labeled 50 nM idInlB variants. The normalized thermophoresis value Fnorm% was plotted against the gC1qR concentration. Data of one from three independent measurements are shown. (**D**) Mean values of dissociation constants (K_d_) ± standard deviation for human gC1qR binding with InlB variants from three independent experiments.

Next, dissociation constants for the alternative InlB receptor, gC1qR, were analyzed. The human gC1qR was titrated in a concentration range from 0.076 nM to 2.5 μM against fluorescent-labeled 50 nM isoforms of InlB. The normalized thermophoresis value Fnorm % was plotted against the idInlB_CC1_, idInlB_CC7_, and idInlB_CC9_ concentration fitted to a 1:1 binding model resulting in a dissociation constant Kd = 7.4 ± 0.8 nM, 10.2 ± 0.9 nM, and 21.5 ± 1.0 nM, respectively (Figure 2A–D). These data showed that three variants bound the receptor, but idInlB_CC9_ bound it three times weaker than idInlB_CC1_ and it two times weaker than idInlB_CC7_. There was no significant difference in binding strength between the idInlB_CC1_ and idInlB_CC7_ isoforms for gC1qR. Note that the binding curves of idInlBs with gC1qR and with c-Met have opposite forms. The microscale thermophoresis method is based on changes in fluorescence of the ligand stained with the fluorescent dye. Upon receptor binding, fluorescence of the ligand can increase or decrease in dependence on a receptor and a buffer composition. If the ligand–receptor interactions increase fluorescence, the curve is rising; if these interactions decrease fluorescence, the curve is descending. The calculation of the Kd values is determined from the shape of the curve and does not depend on its direction [44].

### 2.2. The CC1-Specific InlB_CC1_ Improves Bacterial Multiplication in Macrophages Comparatively to Other InlB Isoforms

**Figure 3:**
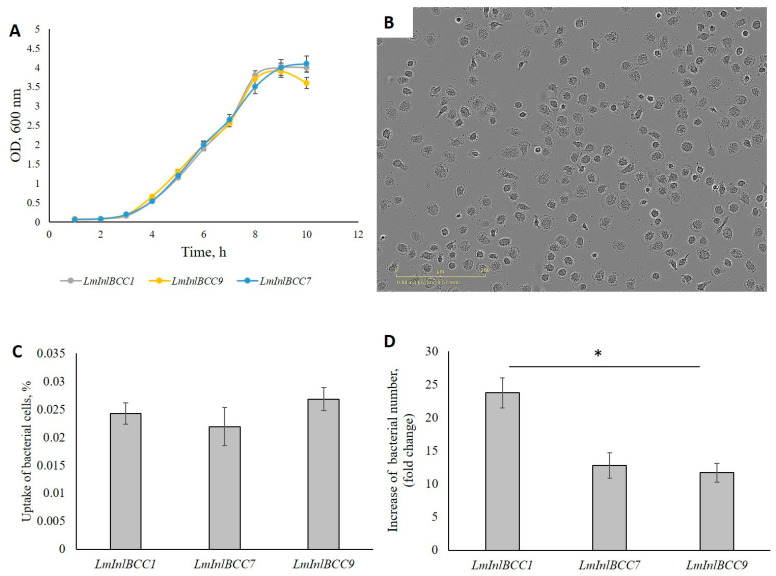
Interaction of M1-phenotype macrophages with isogenic strains of *L. monocytogenes*. Bacteria were added to the macrophages in MOI 1:100, incubated for 1 h. To destroy extracellular bacteria, gentamicin was added at a concentration of 100 µg mL^−1^ and incubated for 1 h. To assess the effectiveness of bacterial capture, macrophages were washed three times with PBS and 1% triton X-100 were lysed. In the analysis of intracellular reproduction, the medium was replaced with a supportive one with the addition of gentamicin 20 µg mL^−1^ and after 24 h macrophages were washed three times with PBS and 1% triton X-100 were lysed. After cell lysis, serial dilutions were made. (**A**) Growth curves of three isogenic strains *LmInlBCC1*, *LmInlBCC7* and *LmInlBCC9*; (**B**) Morphology of cells derived from blood monocytes of healthy donors. The cells have characteristic outgrowths on the cell surface and a large rounded nucleus. The photos were obtained using the IncuCyte^®^ S3 Live Cell Imaging System (Sartorius; Göttingen, Germany); (**C**) The uptake of isogenic recombinant strains of *L. monocytogenes* into M1-phenotype macrophages. The f-ratio value is 2.63602. The *p*-value is 0.104461. The result is not significant at *p* < 0.05 *(n* = 4); (**D**) An increase in the number of bacteria of isogenic strains of *L. monocytogenes* in macrophages of M1-like phenotype within 24 h after infection (* the *p*-value between *LmInlBCC1* and *LmInlBCC7/LmInlBCC9* is <0.05, *n* = 4).

Isogenic *L. monocytogenes* strains previously constructed on the basis of the strain EGDeΔinlB [12] were used. The strains *LmInlBCC1*, *LmInlBCC7*, and *LmInlBCC9* expressed full-sized InlBs that differed in idInlB domain but possessed the same carboxy-terminal GW domains and expressed from the same *inlAB* promoter at a very similar level (Supplementary Material See Figure S2). All three strains were characterized by the same growth rate under standard cultivation conditions (Figure 3A). According to the cell growth curves, the equations of curves of the form y = y_0_∙e^µt^ were found. In accordance with the equation, the specific growth rate µ was calculated. The specific growth rates were 0.426 ± 0.004 h^−1^ for all three recombinant strains.

To analyze bacterial uptake and further intracellular fate, human M1-phenotype macrophages derived from blood monocytes as described in Materials and Methods were used (Figure 3B) [39]. M1 macrophages are pro-inflammatory macrophages aimed at the elimination of pathogens in the course of infection, and their role in the elimination of *L. monocytogenes* is well-established [33].

The isogenic recombinant *L. monocytogenes* strains were added to macrophages with the multiplicity of infection MOI of 100 bacteria/host cell; gentamicin was added 1 h later to remove extracellular bacteria. Intracellular bacteria were enumerated 1 h and 24 h after gentamicin addition to evaluate the efficiency of the uptake and intracellular bacterial multiplication. All recombinant strains demonstrated similar values of the uptake by macrophages (the f-ratio value is 2.63602, the *p*-value is 0.104461. The result is not significant at *p* < 0.05., Figure 3C). Meantime, after 24 h, the number of intracellular *LmInlBCC1* bacteria was twice as high as that of *LmInlBCC7* and *LmInlBCC9* bacteria (the f-ratio value is 7.51909, the *p*-value is 0.005472, Figure 3D). These results suggested that InlBCC1 provided better bacterial infection within macrophages.

### 2.3. Exogenic idInlB Affects Bacterial Uptake and Proliferation in Macrophages in an Isoform-Specific Manner

It was suggested that differences in the proliferation of the isogenic strains within macrophages might be due to differential interactions of InlBCC1 and InlBCC7/InlBCC9 isoforms with their target receptors, presented on the macrophage surface. To obtain data in favor of this suggestion, macrophages were pretreated with distinct idInlB isoforms, and the levels of bacterial uptake and proliferation in macrophages were detected. The InlBCC1, InlBCC7, and InlBCC9 strains differed only by idInlB, so using distinct idInlBs corresponded to distinctions between strains. Further, such an approach allowed for dissecting the effects of the secreted and bacterial cell-wall-bound InlB forms. idInlB lacks peptidoglycan-binding GW-domains of the full-length InlB and therefore the exogenic proteins were soluble and could not bind to the bacterial surface. idInlB_CC1_ (lineage I specific InlB isoform) and idInlB_CC7_ (lineage II specific isoform) taken in a concentration of 1 µg/mL were used. This concentration represented levels of cell-free InlB accumulated in supernatants of isogenic strains grown overnight (See Supplementary Material Figure S2).

**Figure 4:**
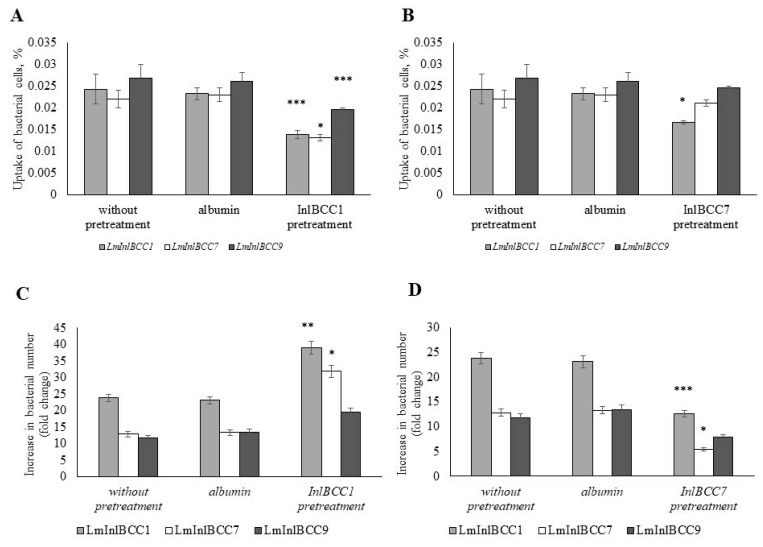
Interaction of M1-phenotype macrophages with isogenic strains of *L. monocytogenes* after pretreatment with a soluble form of InlB. Macrophages were incubated with soluble isoforms of idInlBs for 15 min. Macrophages were washed three times with PBS. Bacteria were added to the macrophages in MOI 1:100, incubated for 1 h. To destroy extracellular bacteria, gentamicin was added at a concentration of 100 µg mL^−1^ and incubated for 1 h. To assess the effectiveness of bacterial capture, macrophages were washed three times with PBS and 1% triton X-100 were lysed. In the analysis of intracellular reproduction, the medium was replaced with a supportive one with the addition of gentamicin 20 µg mL^−1^ and after 24 h macrophages were washed three times with PBS and 1% triton X-100 were lysed. After cell lysis, serial dilutions were made. The addition of human albumin at a concentration of 1 µg mL^−1^ was used as a control. (**A**) The uptake of isogenic recombinant strains of *L.monocytogenes* into M1-phenotype macrophages with pretreatment idInlB_CC1_ (*** *p* < 0.001, * *p* < 0.05, *n* = 4); (**B**) The uptake of isogenic recombinant strains of *L.monocytogenes* into M1-phenotype macrophages with pretreatment idInlB_CC7_ (* *p* < 0.05, *n* = 4); (**C**) An increase in the number of bacteria of isogenic strains of *L. monocytogenes* in macrophages of M1-like phenotype with pretreatment idInlB_CC1_ within 24 h after infection (** *p* < 0.01, * *p* < 0.05, *n* = 4); (**D**) An increase in the number of bacteria of isogenic strains of *L. monocytogenes* in macrophages of M1-like phenotype with pretreatment idInlBCC1 within 24 h after infection (*** *p* < 0.001, * *p* < 0.05, *n* = 4).

Pretreatment with both idInlB isoforms decreased the uptake of all three recombinant *L. monocytogenes* strains (Figure 4A). The effect on macrophage activity was dependent on the idInlB isoform, and idInlB_CC1_ affected bacterial uptake more strongly than the idInlB_CC7_ isoform (see Figure 4A). However, the effect on the bacterial uptake was dependent not only on the idInlB isoform used for pretreatment but also on the InlB isoform carried by the bacterial strain. Thus, the uptake of the strain carrying InlBCC9 was poorly affected by pretreatment with the soluble idInlB, especially with idInlB_CC7_, suggesting that surface-bound and/or its own secreted InlB can interfere with processes started by soluble idInlBs.

The proliferation of captured bacteria within macrophages was also affected by pretreatment with soluble idInlBs but idInlB isoforms provided different effects on bacterial multiplication within macrophages. idInlB_CC1_ improved the proliferation of all three tested recombinant strains, with the best effect observed for the LmInlBCC1. In contrast, idInlB_CC7_ declined in intracellular proliferation.

Thus, the results demonstrated that pretreatment with soluble idInlB_CC1_ noticeably disturbed macrophage functions decreasing pathogen uptake and improving its intracellular multiplication, while idInlB_CC7_ affected only bacterial uptake. These data were in line with observations made with isogenic *L. monocytogenes* strains (Figure 3). The observed interference of exogenic idInlBs with InlBs carried by strains further supported the role of InlB variability in interactions of *L. monocytogenes* with macrophages. On the whole, the obtained results supported the suggestion about the effect of secreted InlB forms on *L. monocytogenes* survival.

Obtained results suggested that soluble InlBCC1 decreases bacterial uptake by macrophages while soluble InlBCC7 does not. Indeed, pretreatment with soluble InlBCC1 decreased bacterial uptake by macrophages whatever strain was used, while pretreatment with soluble InlBCC7 did not affect the uptake of strains carrying InlBCC7 and InlBCC9 (Figure 4A). The decreased uptake of the strain carrying InlBCC1 after pretreatment with InlBCC7 could be due to a heterogeneous dimerization of exogenously added InlBCC7 and InlBCC1 released from the surface of the LmInlBCC1 strain. The uptake diminishing effect of this combination was less pronounced than the effect of exogenous InlBCC1 and surface released InlBCC7 (i.e., the effect of pretreatment with soluble InlBCC1on LmInlBCC7 uptake) because of the molar excess of the exogenous InlB isoform over the surface released InlB isoform. The concentration of the exogenous InlB isoform was correspondent to the amount of InlB accumulated in the overnight *L. monocytogenes* culture supernatant, while the concentration of the surface-released InlB isoform was less because the culture used for infection was washed before adding to macrophages and only freshly released InlB took part in interactions. The increased bacterial survival after macrophage pretreatment with soluble InlBCC1 suggested that the InlBCC1 isoform but not InlBCC7 affected not only bacterial uptake but also other macrophage functions, including the ability to support the multiplication of intracellular bacteria. The pronounced effect of the InlBCC1 isoform of macrophage functions appeared due to its better interactions with InlB target receptors, and particularly with c-Met (see Figure 1 and Figure 2). The InlBCC1 effect on macrophage functions might play a role in the course of *L. monocytogenes* infection because macrophages represent the first line of the immune defense. In particular, the described impact of InlB_CC1_ on human macrophages might be important for the high virulence of the *L. monocytogenes* strains belonging to the clonal complex CC1, all of which carry the InlB_CC1_isoform [8,14].

## 3. Discussion

This work demonstrated that the secreted cell-free form of the *L. monocytogenes* virulence factor InlB could affect the uptake of *L. monocytogenes* by macrophages and intracellular bacterial proliferation. InlB’s effects on macrophage activity depended on the InlB isoform. InlB, specific to the highly virulent lineage I clone CC1, decreased bactericidal macrophage effects, suppressing bacterium uptake and improving bacterial intracellular multiplication, while the alternative isoform, characteristic for lineage II clones of medium virulence provided a mild effect on bacterial uptake and did not improve and even worsened intracellular multiplication. *L. monocytogenes* uses glucose-1-phosphate (G1P) under the strict positive control of PrfA, the central regulator of listerial virulence, and this metabolic pathway is activated in eukaryotic cells [45]. The lack of energy substrates leads to a decrease in the growth rate of bacteria. It is known that M1-phenotype macrophages actively carry out glycolysis [46]. Therefore, we could assume that the change in the number of bacteria inside macrophages is due to the lack of energy substrates.

However, the obtained results showed that the effects were due to InlB interactions with surface receptors recognized by the internalin domain idInlB and correlated with the efficiency of idInlB interactions with the tyrosine kinase receptor c-Met. In particular, better binding of c-Met by idInlB_CC1_ could be an important factor that provided the observed decrease in bactericidal activity in macrophages treated with this idInlB isoform.

The receptor tyrosine kinase c-Met is best known as an oncogene involved in diverse cancer types [47]. c-Met and its physiological ligand HGF (hepatocyte growth factor) play an important role in embryogenesis. In adults, the HGF/c-Met pathway is involved in wound healing and tissue regeneration, and the HGF/c-Met controlled signaling pathways are involved in the regulation of development, maturation, differentiation, and functions of immune cells [48]. In M1 macrophages, HGF/c-Met signaling was shown to induce the secretion of anti-inflammatory cytokines IL-10 and TGF-β1 and downregulate pro-inflammatory iNOS, TNF-α, and IL-6 via the activation of the PI3K kinase signaling pathways [49,50,51]. The PI3K/Akt/NF-κB signaling plays an important role in macrophage M1/M2 transition and shifts M1 macrophages toward an M2-like phenotype [52]. InlB is a c-Met ligand functionally similar to HGF [53]. The phylogenetically defined idInlB isoforms differentially activate PI3K- and Erk1/2-kinase pathways [13]. In particular, in human epithelial HEp-2 cells, idInlB_CC1_ more efficiently activates the PI3K/Akt-signaling pathway and less efficiently activates the Erk1/2 signaling pathway if compared with idInlB_CC7_ and idInlB_CC9_. Thus, the obtained results suggested that idInlB_CC1_ could induce M1/M2 transition in macrophages in the same way as HGF does, which in turn can explain the observed decrease in anti-bacterial macrophage activity.

Macrophages are principal cells of the innate immune response responsible for bacterial killing. The task of macrophages is to neutralize bacteria trapped in the phagosome by producing reactive oxygen species. However, in the course of evolution, some microbes have developed mechanisms that allow them to evade the immune response such as antigenic variability, the production of substances blocking the development of the immune response, avoiding phagocytosis, and/or inhibiting the maturation of the secondary phagosome. The key mechanism used by *L. monocytogenes* to avoid being killed in a macrophage’s phagosomes is incomplete phagocytosis and the escape of bacteria into the cytosol due to the activity of the pore-forming cytolysin listeriolysin O [37]. Another mechanism used by *L. monocytogenes* includes modulation of Type I interferon production, which contributes to the launch of apoptosis of macrophages [54]. Mansell et al. demonstrated that InlB activated the transcription factor NF-kB in J774 macrophages [55]. The obtained results that demonstrated a correlation between the efficiency of the idInlB isoform interactions with c-Met and the effects of this isoform on macrophage antibacterial activity are in line with these data because c-Met-controlled PI3K/Akt signaling pathways are central to NF-κB regulation [52,56,57].

The obtained results revealed a novel role of InlB in *L. monocytogenes* virulence. InlB is an established virulence factor that allows *L. monocytogenes* to invade non-professional phagocytes [58]. Previously, the authors of the present paper demonstrated that phylogenetically determined InlB isoforms affected the invasion of isogenic *L. monocytogenes* strains into epithelial cells in a host- and/or tissue-specific manner [12,17,18]. Here, it was demonstrated that InlB from the highly virulent CC1 clonal complex impaired the function of human macrophages to capture and degrade *L. monocytogenes*. The fact that InlB_CC1_ was able to suppress macrophage function correlates with epidemiological data on CC1 belonging to the most virulent clonal complexes for humans [15]. Suppression of macrophage function directly affects the virulence of microorganisms, since it disarms the first line of defense and allows for more efficient reproduction in the tissues of the macro-organism. Strategies employed by some pathogenic bacteria to survive in hostile environments of the host include reprogramming macrophages to the M2 subtype. *Salmonella* was shown to use the SPI1 effectors for the epigenetic reprogramming of macrophages responsible for bacterial long-term survival [59]. M2 polarization was shown to favor *Staphylococcus aureus* survival and further infection [60]. Obtained results suggested that this strategy was used by *L. monocytogenes* strains belonging to highly virulent clones.

On the whole, the obtained results demonstrated that *L. monocytogenes* possessed mechanisms allowing the modulation of macrophage activity. The efficiency of these mechanisms depends on phylogenetically determined InlB variability, which in this way can affect the severity of infection. These results confirm the assumption that the selection of certain dominant InlB isoforms contributes to the formation of highly virulent clonal complexes.

## 4. Materials and Methods

### 4.1. In Silico Analysis

The sequences of proteins were compared with those available in GenBank using Basic Local Alignments Tool (BLAST) analysis. Allelic numbers of InlB were determined using the *L. monocytogenes* MLST database (https://bigsdb.pasteur.fr/listeria/listeria.html (1 August 2021)). Sequences were proofread and assembled in Unipro UGENE version 35.1 (http://ugene.net/ (3 August 2021)). Protein alignment was performed using Clustal W. To assess the matching of the distance between multiple sequence alignments, the Unipro UGENE 39 software was used.

### 4.2. Purification of idInlBs

*E. coli* BL21 strains encoding recombinant idInlBs (Table 1) were concentrated by centrifugation, resuspended in the buffer A (30 mM imidazole, 150 mM NaCl, 10 m Na_2_PO_4_, pH 7.4) and disrupted by ultrasonic disintegration. Following this, cell debris was sedimented by centrifugation, and the supernatant was collected. The supernatant was applied to the HisTrapTM FF Crude column (Sigma Aldrich, St. Louis, MO, USA). The column was washed with 5 volumes of buffer A, and then the protein was eluted with the buffer B (500 mM imidazole, 150 mM NaCl, 10 mM Na_2_HPO_4_, pH 7.4). The purified protein was dialyzed against PBS. The final protein concentration was determined by the Bradford method. The purified protein was stored at −20 °C.

### 4.3. Assessment of the Dissociation Constant of InlB Interactions with the Target Receptors

The capacity of natural idInlB isoforms to bind the c-Met and gC1qR was measured with microscale thermophoresis (MST) (Jerabek-Willemsen, Wienken, Braun, Baaske, and Duhr, 2011). The chimeric protein HGFR/Fc (H0536, Sigma-Aldrich, USA) and gC1qR (Invitrogen, #11874H08E25, Waltham, MA, USA) were used to analyze the binding of natural isoforms of idInlB to eukaryotic receptors. The isoforms of idInlB-His-Tag were stained with RED-tris-NTA according to the protocol. The concentration of idInlB-His-Tag was kept constant at 50 nM while twofold dilutions of the unlabeled receptors c-Met and gC1qR in the PBST (PBS supplemented with 0.05% Tween-20) buffer ranged between 0.076 nM and 2.5 μM. Samples were loaded into Monolith NT.115 Premium Capillaries, and MST analysis was performed using the Monolith NT.115 system (Nano Temper Technologies GmbH, München Germany). The LED/excitation power was 80%, and the MST power was 40%. Data analysis was performed using the MO. Affinity Analysis software v. 2.3.

### 4.4. Isolation of Mononuclear Cells from Blood and Differentiation of Human Macrophages

Human macrophages were differentiated from peripheral blood monocytes. Monocytes were isolated from healthy donors using Ficoll-Paque Premium (GE Healthcare, Chicago, IL, USA) and the adherence method [62]. Monocytes were incubated in RPMI-1640 medium supplemented with 2% heat-inactivated human AB serum, 2 mM L-glutamine, 10 mM HEPES, 50 µM β-mercaptoethanol, 2 mM sodium pyruvate, and 2 mM MEM Vitamin (HyClone, Uhta, UT, USA) at 37 °C and 5% CO_2_ for 6 days. On the 4th day, the medium was fully refreshed. On the first and fourth days, 50 ng/mL GM-CSF (Sci-Store, Moscow, Russia) was added to the medium. Cells were stained with fluorophore-conjugated primary antibodies against CD11b (APC-Cy7), CD80 (PE-Cy5), CD86 (BV421), HLA-DR (PE-Cy7) and analyzed by a flow cytometer (Navios, Beckman Coulter, Inc., Brea, CA, USA).

### 4.5. Bacterial Strains and Growth Conditions

*L. monocytogenes* was cultivated in the BHI medium (Becton, Dickinson and Company, Franklin Lakes, NJ, USA) and grown at 37 °C with agitation at 200 rpm. Plasmid-bearing strains were grown in the presence of 10 µg mL^−1^ erythromycin (Sigma-Aldrich, USA) to maintain the plasmid. To prepare a culture for infection, bacteria were grown to the mid-exponential phase, washed with PBS (Amresco, Cochran Rd, Solon, OH, USA) three times, aliquoted, and frozen in the presence of 10% glycerol (Sigma-Aldrich, USA). The growth rate was estimated using its optical density at 600 nm. The strains were grown overnight in BHI medium (Becton, Dickinson and Company, USA) at 37 °C and subsequently diluted 1:100 (the volume of night culture: the volume of the fresh media) in the same media. Optical density was measured every hour on an Ultrospec™ 10 Cell Density Meter spectrophotometer (Biochrom, Cambridge, UK). *E. coli* BL21 strains encoding recombinant idInlBs were grown in the LB medium complemented with 100 µg mL^−1^ kanamycin and 1 mM IPTG.

### 4.6. Estimation of InlB Concentrations by ELISA

*L. monocytogenes* were grown on BHI and BHI supplemented with 0.2% activated charcoal to activate the PrfA-regulon overnight. Bacteria were pelleted by centrifugation (4200 rpm, 15 min). Following this, supernatant and cells were separately used to quantify InlB concentrations. Surface-bound InlB was detected using ELISA. Cells were washed three times with a phosphate-salt buffer and resuspended in 500 µL of carbonate-bicarbonate buffer (pH 9.6). One hundred µL of the resulting suspension was added to a well of the 96-well plate and incubated overnight at +4 °C. Following this, wells were washed with 250 µL TTBS three times. One hundred microliters of HRP-conjugated InlB-specific antibody (Kalinin et al., 2023 [63]) taken in a 1:4000 dilution were added, and the plate was incubated at room temperature for 1 h. Following this, wells were washed as described above, and HRP activity was detected by incubation with the 100 µL TMB substrate. The reaction was stopped by 2 M H_2_SO_4_. Optical density was measured using an iMark microplate absorption reader (Bio-Rad; Hercules, CA, USA) at 450 nm.

A sandwich ELISA was used to measure secreted InlB concentrations in the overnight culture supernatants. Wells of the 96-well plates were filled with 100 µL of InlB-specific antibodies (4 µg/mL) (Kalinin et al., 2023 [63]) and incubated overnight. After that, the wells were washed with TTBS, sample supernatants were added and incubated at room temperature for 1 h. Following this, wells were washed with TTBS, and HRP-conjugated InlB-specific antibodies were added (1:4000, 100 µL per well). After that, the wells were washed six times with TTBS. The signal was detected with a TMB substrate (Thermo Fisher Scientific, Waltham, MA, USA) added in the amount of 100 µL per well; the reaction was stopped by adding 100 µL of 2 M H_2_SO_4_. Optical densities at 450 nm were measured using an iMark spectrophotometer (Bio-Rad, California, CA, USA). The InlB concentration was determined using a calibration curve and recalculated by the number of CFU (colony-forming units) in the sample.

### 4.7. Infection of Human Macrophages and Analysis of Bacterial Multiplication in Macrophages

Human macrophages were obtained as described above and grown in 24-well plates with up to 80,000 cells per well. Following this, the nutrient medium was replaced. Fresh medium or fresh medium supplemented with soluble forms of idInlB_CC1_ and idInlB_CC7_ was taken in a concentration of 1 µg mL^−1^. The addition of human albumin (SCI-store, Moscow, Russia) at a concentration of 1 µg mL^−1^ was used as a control. Cells were incubated for 15 min. following this, samples were washed three times with PBS. Bacterial suspension in pre-warmed DMEM was added so that the multiplicity of infection (MOI) was 100 CFU per macrophage. The cells were incubated for 1 h at 37 °C and 5% CO_2_. After incubation, the cells were washed three times with PBS. Then fresh DMEM (Paneco, Moscow, Russia) supplemented with 100 µg mL^−1^ gentamicin (Sigma-Aldrich, St. Louis, MO, USA) was added, and cells were incubated for 1 h more. The cells were washed three times with PBS and cell lysates were obtained after 2 min incubation with 1% Triton X-100 (Sigma-Aldrich, St. Louis, MO, USA). Intracellular bacteria were enumerated by plating serial dilutions of cell lysates, and colonies were counted after 24 h incubation.

Macrophages were obtained and infected as described above. After 1 h incubation with gentamicin, cells were washed three times with PBS, and fresh DMEM supplemented with 20 µg/mL gentamicin was added. Cells were incubated for 24 h at 37 °C and 5% CO_2_. Cells were washed three times with PBS and cell lysates were obtained after 2 min incubation with 1% Triton X-100. Intracellular bacteria were enumerated by plating serial dilutions of cell lysates, and colonies were counted after 24 h incubation.

### 4.8. Statistics

Values are expressed as mean ± SD. Statistical analysis was performed using a one-way ANOVA with post hoc Tukey’s test. Statistical differences were considered significant when the *p*-value was <0.05.

## Figures and Tables

**Table 1:** Strains used in the work.

Species/Strain	Characteristics	Reference
*L. monocytogenes*		
EGDeΔinlB ^1^	EGDe derivative with inlB gene deletion, clonal complex CC9, lineage II	[61]
EGDeΔinlB::InlBCC1	EGDeΔinlB supplemented with the pInlB9 plasmid, idInlB clonal complex CC1, lineage I	[17]
EGDeΔinlB::InlBCC7	EGDeΔinlB supplemented with the pInlB14 plasmid, idInlB clonal complex CC7, lineage II	[17]
EGDeΔinlB::InlBCC9	EGDeΔinlB supplemented with the pInlB13 plasmid, idInlB clonal complex CC9, lineage II	[17]
*E. coli*		
BL21 (DE3)	F–ompT gal dcm lon hsdSB(rB-mB-) λ(DE3 [lacI lacUV5-T7 gene 1 ind1 sam7 nin5])	NewEngland BioLabs
BL21::pET28b(+)::idInlB_CC1_	BL21 supplemented with the pET28b(+)::idInlB_CC1_ plasmid	[13]
BL21::pET28b(+)::idInlB_CC7_	EGDeΔinlB supplemented with the pET28b(+)::idInlB_CC7_ plasmid	[13]
BL21::pET28b(+)::idInlB_CC9_	EGDeΔinlB supplemented with the pET28b(+)::idInlB_CC1_ plasmid	[13]

^1^ The strain was generously provided by Professor J. A. Vazquez-Boland, University of Edinburgh.

## Data Availability

All dates are ethically sound and meet industry-recognized standards.

## Supplementary Materials

The following supporting information can be downloaded at: https://www.mdpi.com/article/10.3390/ijms24087256/s1. References [64,65,66,67,68,69] are cited in the supplementary materials.
