# Supplementary material for: Natural Isoforms of Listeria monocytogenes Virulence Factor Inlb Differ in c-Met Binding Efficiency and Differently Affect Uptake and Survival Listeria in Macrophage
Source: Int J Mol Sci. 2023 Apr 14;24(8):7256. doi: 10.3390/ijms24087256 (PMC10139187; doi:10.3390/ijms24087256)
Supplement: Supplementary file 1 [file ijms-24-07256-s001.zip › ijms-2295680-supplementary.pdf]

Supplementary material

To activate macrophages, soluble forms of internal domains of InlB (idInlB$_{CC1}$ and idInlB$_{CC7}$, respectively) were used. To assess the role of natural variants in interaction with macrophages, 3 recombinant strains were selected that carried full-size InlB and differed only in the amino acid sequence in the region of the internalin domain (Figure S2A). The multiple alignment distance matrices within idInlB showed that the convergence between phylogenetic lines was 94% (Figure S2B). There was a difference of 13 a/k substitutions between CC1 and CC7. The structure of internalin B predicted using an online service RaptorX Contact Prediction Server (http://raptorx.uchicago.edu/) (Figure S2C).

A

	N-cap			LRR								Ig-like			
	41	49	69	73	91	117	132	164	176	181	197	205	246	251	262
idInlB$_{CC1}$	**P**	**P**	**L**	**S**	**I**	**A**	**I**	**L**	**L**	**I**	**E**	**S**	**S**	**M**	**I**
idInlB$_{CC7}$	**S**	**P**	**A**	**N**	**V**	**T**	**I**	**P**	**I**	**V**	**Q**	**A**	**P**	**S**	**T**
idInlB$_{CC9}$	**P**	**S**	**A**	**N**	**V**	**A**	**V**	**P**	**I**	**V**	**Q**	**A**	**P**	**S**	**T**

B **Multiple Sequence Alignment Distance Matrix**

	idInlB$_{CC1}$	idInlB$_{CC7}$	idInlB$_{CC9}$
**idInlB$_{CC1}$**	**100%**	**94%**	**94%**
**idInlB$_{CC7}$**	**94%**	**100%**	**99%**
**idInlB$_{CC9}$**	**94%**	**99%**	**100%**

C

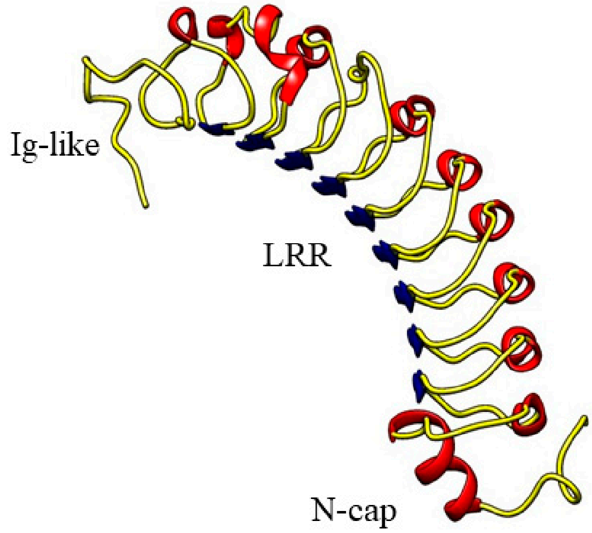

Figure S1. Divergence of naturally occurring variants of the InlB internalin domain. (**A**) Amino acid substitutions. The idInlB$_{CC9}$ sequence was taken as a basis. Substitutions between lineages I and II idInlBs (internalin domain) are shown in bold. LRR for leucine rich repeat (amino acids 41-262 N-cap, LRR and Ig-like domains); (**B**) To assess the matching of the distance between multiple sequence alignments, the Unipro UGENE software was used (http://ugene.net/); (**C**) The structure of the internaline domain InlB (N-cap, LRR and Ig-like domains).

Determination of the InlB level in recombinant strains

InlB, when present on the bacterial surface, mediates the entry into the host cell by zipper-type phagocytosis. InlB interacts with the extracellular domain of Met through its LRR domain, but the full-length protein is required for maximal activation [22]. It has been confirmed that the level of internalin B between recombinant strains is comparable, since interaction with the receptor may depend not only on the isoform of internalin B but also on the concentration of protein. For analysis, strains were grown in the environment of the BHI and BHI with activated charcoal. Positive Regulatory Factor A (PrfA) is the major transcriptional regulator of virulence gene expression in *L. monocytogenes* [64,65]. It has been widely noted that *L. monocytogenes* PrfA-regulated genes are expressed at lower levels in complex media such as BHI or LB broth than in chemically defined media [66,67]. Activated charcoal is commonly used to induce the expression of PrfA-regulated genes in complex growth media [68,69]. *L. monocytogenes* was grown both in a media

without charcoal and with charcoal for activated PrfA-regulon. To analyze the InlB level in recombinant strains, cell cultures were grown to an optical density of 1.8 into BHI with charcoal and without them. The number of bacteria was controlled by seeding on solid nutrient media. The level of InlB on the cell surface and in the supernatant was evaluated using the ELISA test system. See "Materials and methods". In the absence of activated carbon, the InlB level was comparable for three recombinant strains and averaged 98 ± 2.7 ng per ml on the cell surface and 599± 25 ng per ml in supernatant (Figure S2A,B). On the medium with the addition of activated charcoal, the level of InlB for both strains increased to an average of 209 ± 12.8 ng per ml on the cell surface. The level of InlB in the supernatant was different for both isogenic recombinant strains (Figure S2 A,B).

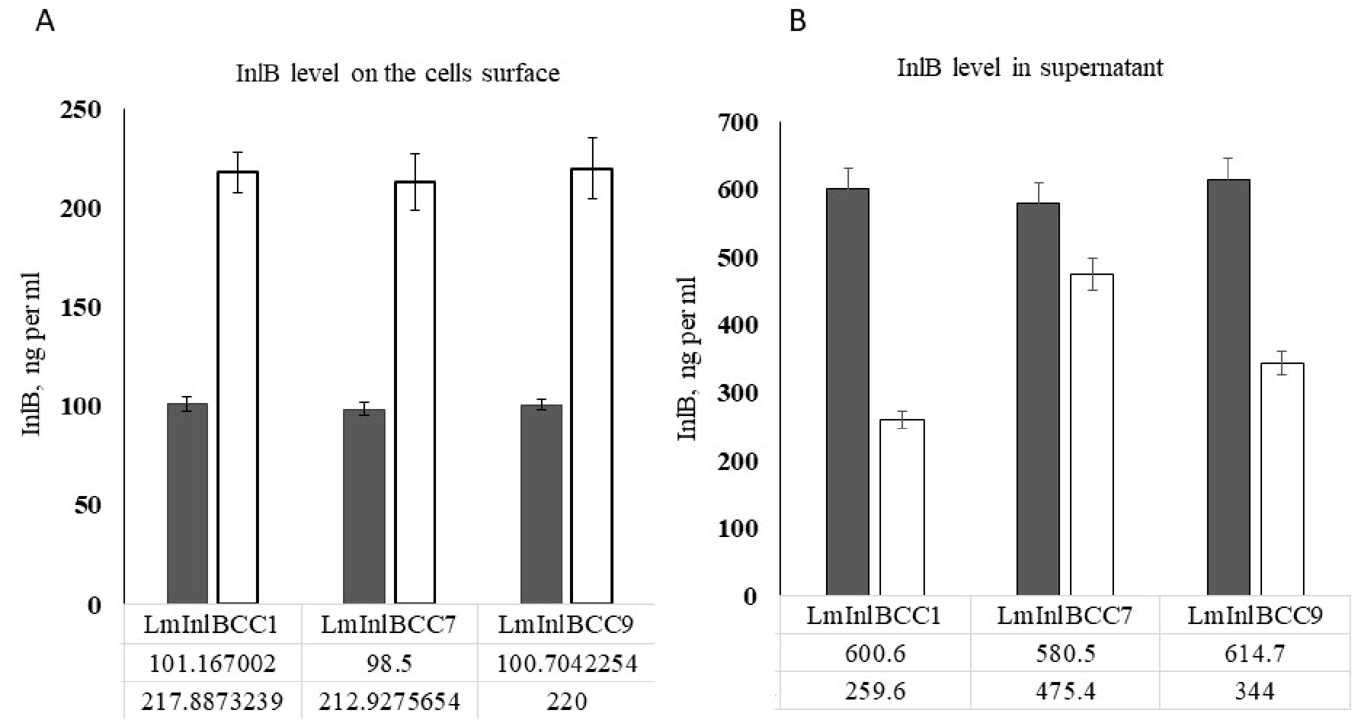

Figure S2. InB level on the surface of bacterial cells. The gray columns correspond to the InlB level when bacteria grow on the BHI medium. The white columns correspond to the level of InlB during the growth of bacteria on the BHI medium with the addition of activated charcoal ($p<0.05$, $n=3$).
